# Medical and Philosophical Intelligence

**Published:** 1812-08

**Authors:** 


### MEDICAL AND PHILOSOPHICAL INTELLIGENCE.

ROYAL SOCIETY.—In a paper by Mr. John Davy, the results of some experiments on silicated fluoric gas, and fluoboric gas, were given; and he described a new and more simple method of procuring this last gas, namely, by distilling together a mixture of dry boracic acid, fluor spar, and oil of vitriol. He stated the specific gravity of these gases, and gave an account of their combinations with ammonia. Fluoboric gas combines in three proportions with ammonia, and with the largest proportions of the alkaline gas forms saline combinations, fluid at the common temperatures of the atmosphere.

A paper by Sir H. Davy was read, on some combinations of phosphorus and sulphur, and on other objects of themical inquiry.

In this paper, Sir H. Davy described pure phosphorous acid as a solid crystalline body, volatile at a moderate degree of heat. He described a combination of this acid with water, likewise a crystalline solid, very combustible, and when decomposed by heat affording a peculiar elastic fluid absorbable by water, not spontaneously inflammable, and consisting of phosphorus united to two volumes of hydrogen condensed into the space of one volume, and which he proposes to call *hydrophosphoric* gas.

He entered into the detail of some experiments on sulphuric acid, which, he stated, cannot exist independently of the presence of water; and he described a solid compound of nitrous acid gas, sulphurous acid gas, and water. He considered all the facts advanced in this paper as affording confirmations of the theory of definite proportions; and he drew some general conclusions respecting the importance of water as a chemical agent. Most of the bodies called *oxides*, when precipitated from aqueous solutions, are, in truth, (said Sir H. Davy) hydrats; and their colors and their properties depend upon the combined water.

*Russell Institution.—May.* In the fourth Lecture Mr. Bakewell proceeded to describe the stratified rocks containing rock-salt and coal. The coal districts in England and in other parts of the world, he said, were generally separated from the compact limestone which contains metallic veins, by thick beds of coarse grit-stone and sandstone, in which some vegetable remains first made their appearance. In the midland counties of England there are two kinds of rock interposed between the coal and the lime, forming together a mass of three hundred yards in thickness. The lower bed consists of a dark reddish reddish-brown shale, in which strata of micaceous sand-stone, and beds of dark limestone, occasionally occur. The upper rock was called by Mr. Whitehurst mill-stone grit, from its containing beds of hard siliceous grit-stone, used for mill-stones. This rock varies both in color and quality in different parts. It was first observed by Mr. Whitehurst, that under this rock no coal is ever found. This observation Mr. Bakewell said was correct, as applied to workable coal, or such beds that were of sufficient thickness to be got with profit; but very thin seams of coal, as well as vegetable impressions, are not unfrequent in these rocks. They extend over a considerable part of the northern counties, and form the range of central hills from the north of Derbyshire to Craven in Yorkshire. Mr. Bakewell said he was inclined to believe that the lower bed, a dark-brown shale, changed its quality as it passed into Cheshire, and was there the red sand-rock of that and the adjacent counties. He observed that he did not consider some difference of external character alone, sufficient to disprove the identity of strata which may spread over a large tract of country. In this red sand-rock the rock-salt of Cheshire is found. He described the various repositories of salt in Spain and different parts of the world, and observed, that it had been found at the height of 9000 feet above the level of the sea; and from its position, as well as from the difference of its constituent parts from those of sea-salt, he was inclined to believe that it had not been formed by the evaporation of sea-water, as some geologists have asserted. The rock-salt of Cheshire is remarkably free from impurities, containing, according to the correct analysis of Dr. Henry, not the least sulphat of magnesia, and not one grain in 1000 of muriate of magnesia. In the same quantity of sea-salt, not less than 46 grains of these two saline impurities occur.

In the strata over these rocks we meet with beds of coal occupying distinct districts called coal-fields. Mr. B. then described the various kinds of stone-shale, and iron-stone, that alternate with coal, and the appearances which offer indications of its presence. The position into which coal strata are thrown by faults and dykes in different parts of England, were explained by drawings and sections. A most singular elevation of a bed of coal in the vicinity of the red rock in Lancashire was particularly noticed. Where this rock comes nearly in contact with the coal, the stratum is raised up vertically, whilst seven other beds of coal in the same field are all inclined at an angle of 25 degrees. Mr. Bakewell stated, that he had communicated an account of this coal-field to the Geological Society, as he conceived that it might lead to some discoveries respecting the geological relations of the red sand-rock with the coal strata. The obstacles which impede the working of coal-mines, from the fire and choak damps, were described. The nature and properties of these noxious vapours were illustrated by experiments, and some account given of the different modes of clearing the mines in different districts.

Mr. Bakewell observed, that Dr. Miller, and other writers on the coal districts of England, had entirely overlooked the coal of the

West

West Riding of Yorkshire, although the quantity procured there supplied a population nearly equal to that of London, besides the extensive manufactories in that county. He described the manner in which the quantity of coal in the coal-fields of Northumberland and Durham had been estimated; from whence it appeared, that, at the present rate of consumption, they would be entirely exhausted in the space of three centuries. Were there no other repositories but these, he observed that it would become the duty of a wise statesman to prevent exportation to foreign countries, and to plant all our waste lands, and provide against impending, though remote, calamity. Let any person, said he, reflect on the condition of the metropolis were it deprived for three months of the supply of coal. All the wood in the country would be destroyed, our manufactories would be annihilated, and a scene of national calamity would ensue, of which we can scarcely form an idea.

But, besides extensive coal-fields in Yorkshire and other parts of England, at present scarcely touched, there exists a great repository in South Wales of one thousand square miles of coal, which, from the thickness of the different strata, he calculated would supply the consumption of Britain for several thousand years to come, estimating that consumption at twelve million tons annually. He concluded by giving a short outline of the coal districts in different parts of the world, and the periods at which coals appear to have been introduced for fuel. It may excite a smile, said he, at the ignorance of our ancestors, to find an act of parliament in the reign of Elizabeth, prohibiting the burning of coal in London during the sitting of parliament; but probably posterity, at no distant period, may look with equal surprise at the general indifference evinced by landed proprietors of the present day, respecting the mineral substances on their own estates.

---

*A new and certain Method, discovered and proposed, whereby any mistake in the practice of Vaccination may be easily detected, and the use of the genuine Kine Pock rendered more universal.*— Individuals or families residing in any part of the United States, who cannot otherwise procure the most satisfactory proof of their having obtained the genuine Kine Pock, are hereby informed, that if, after submitting themselves to be vaccinated, they will take care of the scabs or crusts which every operation will produce, (whether the same may have been effectual, spurious, or imperfect,) and will send them to the subscriber *for examination*, they may obtain the same evidence of their safety, or continued liability to take the small-pox, as if they had been personally visited by him every day during the whole process of their vaccination.

To obtain this desirable proof of personal safety, however, some little care and attention is requisite on the part of the patient. During the period of inflammation or soreness, the arm into which the infection may have been inserted, should be taken as much care of as possible. The pock, or sore, should not be altered or disturbed by applications of any kind; neither should it be scratched, bruised, or or injured, in any manner whatever: but the disease should be suffered to come to its own natural termination—*a scab or crust will be then formed in due time; and it will be found, on a strict and proper examination, to be perfectly indicative of the disease which produced it, whether the same may have been the genuine Kine Pock, or any of those spurious affections which have been so often mistaken for it.* On the eighth day after the operation, or as soon as the scabs or crusts become loose, they should be taken off, and wrapped in a little fine lint or cotton; they may then be folded up in a small piece of clean white paper, on which must be written the name, age, and place of residence, of the person or persons from whom they were taken; and also the time when they came off.

In all cases submitted as above, where the vaccination has been effectual, a certificate signed by the subscriber will be given, declaring that the person who has been the subject of it will never thereafter be liable to take the small-pox; and, if any vaccination may be found to have been spurious, imperfect, or ineffectual, the necessary precautions to be taken will be particularly and promptly given in a letter of advice.

In making known to the public this new method of correcting the mistakes which have been everywhere so frequent and also so fatal in the practice of vaccination, the subscriber deems it necessary to state, that he has adventured to propose it only after the most mature investigation of its merits. His experience during eleven years nearly past, will now, he believes, fully justify him in declaring, that *there is no possible deception or mistake in vaccination, let the operation be performed by whom, when, or where, it may, which he will not be able to detect with the utmost certainty, by the examination proposed; and he has no doubt but that every other practitioner, who is perfectly acquainted with the character of the genuine kine pock, will soon discover that he can also, with a little attention, judge correctly of the effect of any operation, by an accurate examination of the scabs or crusts, as herein suggested.* He solicits, however, the most strict scrutiny of the utility of his plan, by a discerning public, and, should it fail to accomplish the highly-important objects proposed by him, no one will be more free to acknowledge it, nor yet more deeply to lament it, than himself.

For all communications on this subject, either to or from the subscriber, the medium of the post-office is recommended, as being the most convenient, expeditious, safest, and best. The postage, however, must in all cases be paid, otherwise no communication will be taken from the post-office.

*Baltimore, March* 17, 1812. JAMES SMITH.

---

The council of the Harveian Society, Edinburgh, has announced the following *prize questions* for three years to come.

For 1812. An Experimental Essay on the best method of preparing a Soporific Medicine from the *Lactuca Sativa.*

For 1813. An Experimental Essay on the Effects of the *Succus Spissatus Lactucæ Sativæ* on the Human Body.

For

For 1814. An Experimental Essay on the Effects of the *Digitalis Purpurea* on the circulation of the Blood, both in the human species and other animals, particularly on frogs.

Dissertations on these subjects must be transmitted to Dr. Duncan, senior, secretary to the Society, on or before the 1st of January following the year for which they are announced.

---

The Members of the Royal Medical Society of Edinburgh are invited to write an *Experimental Essay* on the following subject:

"To determine, by experiment, what substances are exhaled by the skin; and the changes, if any, which they produce on the surrounding air."

The dissertations are to be written in English, Latin, or French, and are to be delivered to the secretary, on or before the 1st of December, 1813. The adjudication will take place in the last week of February following.

To each dissertation shall be prefixed a motto, and this motto is to be written on the outside of the sealed packet, containing the name and address of the author. No dissertation will be received with the author's name affixed; and all dissertations, except the successful one, will be returned, if desired, with the sealed packet unopened.

N. Bain, *Sec.*

---

The Jacksonian Prize of the Royal College of Surgeons, in London, for the year 1811, has been adjudged to Mr. Joseph Hodgson, of Bucklersbury, London, for his Dissertation on the Diseases of Arteries and Veins, comprising the pathology and treatment of Aneurism and Wounded Arteries.

---

In the short space of time between the 26th of March and the 2d of May last, a succession of dreadful physical events has demolished some of the finest cities in South America, and shaken several of the islands to their foundations, wasting them with fire, and covering them with ashes and dust. The General commanding the troops of Coro de Domingo Campo Verde, writes to the Governor of the province of Coro, that the city of Barquisimeto was on the 26th of March buried in ruins by a most dreadful earthquake. And he communicates the following moral event, which immediately succeeded. The inhabitants of the district, conceiving the phænomenon to have been an immediate manifestation of divine displeasure for having rebelled against their sovereign, returned to their allegiance. "All the villages," says the General, "belonging to this district and the city of Jouyo, with the greater part of the villages belonging thereto, have come and offered submission under the banners of Ferdinand VII." and subsequently adds, "By the panic and terror with which the inhabitants are struck since the earthquake, I have no doubt that the army of Coro will eventually conquer the province of Venzuela."

During this terrible commotion of the earth, which appears to have broken out at intervals between the 26th and 28th, the following cities were destroyed in the province of Coro: Barquisimeto, totally destroyed; Arilaqua, sunk; Santa Rosa, also sunk; Caudare and Phelipe, destroyed; and St. Charles and Caramaoate, greatly injured. In the province of Caraccas, the same calamity has been equally tremendous. The cities of Caraccas, Victoria, Valencia, Porto Cavello, Laguira, New Barcelona, Maiquetia, and Cumana, have been almost entirely ruined.

The same or similar subterranean causes, which have so severely desolated the continent, about the end of April manifested their influence among the West-Indian islands. We have received details from the West Indies of phenomena probably connected with these awful events on the American continent. Early in May, a volcano in the island of St. Vincent, which had long been quiescent, poured out fire, smoke, and ashes, in immense quantities; accompanied with dreadful explosions. Two of the principal rivers of the island have been dried, and new ones formed; the ashes and stones thrown out are said to be from two to three feet thick on some estates, and the quantity of a lighter kind floating in the air, darkened the atmosphere to an extraordinary degree.

The island of Barbadoes has suffered exceedingly from, possibly, the volcano of St. Vincent. Soon after, as it should seem, that the volcano began to explode in the latter island, a heavy and intense darkness came over Barbadoes, continuing from seven in the morning till the afternoon, accompanied with the sound, as was thought, of distant thunder. During this darkness, an immense quantity of ashes, and which had occasioned the darkness, fell all over the island. The matter which fell, is described as being at first, a large black substance, very coarse, but gradually becoming as fine as Scotch snuff, and covering, in a few hours, the streets and tops of the houses several inches thick.

---

Dr. Merriman will deliver another course of Lectures on Midwifery, and the Diseases of Women and Children, at the Middlesex Hospital, during the months of August and September. The course will commence on Wednesday, August the 5th.

---

A Literary and Philosophical Society has lately been instituted at Liverpool. Its object is to collect information in all branches of literature and science, which is laid before the Society in the form of essays and papers. The number of members already amounts to near sixty, and their meetings are held monthly, from October to May inclusive. The communications and attendance are voluntary. The officers of this Society are, at present, the Rev. Theophilus Houlbrooke, president; Rev. Joseph Smith, Dr. Bostock, and John Theodore Koster, esq. annual vice-presidents; Dr. Thomas Steward Traill, secretary, to whom communications are to be addressed.

---

Dr. Joseph Denman, died suddenly, on the 21st instant, at his house, Chester Place, Vauxhall Road. He was the author of an Essay on the Buxton Waters, and of several small Tracts not in the direct line of his profession; but will be better recognised among his brethren, perhaps, as the elder brother of Dr. Denman of Mount Street.

BOTANICAL

